# A Systematic Treatise

**Published:** 1852-01

**Authors:** 


### BIBLIOGRAPHICAL.

***A Systematic Treatise, Historical, Etiological and Practical, on the Principal Diseases of the Interior Valley of North America, as they Appear in the Caucasian, African, Indian and Esquimaux Varieties of its Population.*** **By Daniel Drake, M. D.**

In a previous notice of this work, we called the attention of our readers to its original character, as its most characteristic feature, and in again alluding to it after a more attentive perusal of its contents, we are constrained to note once more this peculiarity. Dr. Drake, during a long period, devoted to the duties of practicing and teaching the practice of medicine, has been an attentive and patient observer. Fortunately for the cause of medical science, his observations have not been confined to those circumstances immediately connected with the diseases under investigation, but were extended to collateral inquiries, frequently quite remote, but yet having an important bearing upon the modifications of disease.

He was thus led, many years since, to investigate the geological characteristics of the immediate neighborhood of Cincinnati, and at a later period, those of the entire valley bounded upon the one side by the Atlantic, and on the other by the Rocky mountains, and known as the valley of the Mississippi, with especial reference to their effects, in the preservation of health and in the production of disease. The result of these observations clearly showed the important agency exerted by geological phenomena, in the modification of disease, at once curious and interesting, determining its character, fixing its type, and governing its action.

Beneath the soil covering the surface of this extensive valley, and which like that of the loose covering found elsewhere, consists of the debris formed by the accumulations from the disintegration of the subjacent rocks, and which presents characteristic differences dependent upon the rocky formation of which they are composed, beginning at the Gulf of Mexico, which is surrounded by broad and deep alluvial deposits, the valley rises towards the north, into a diluvial, and then into a tertiary formation. To this latter succeeds an extensive cretaceous deposit, extending into West Tennessee, followed by sandstone and shell, which in their turn are succeeded by transition limestone, and are finally terminated upon the north by primitive rocks. In all this arrangement "there is a geological, not less than a geographical, unity in the interior valley. Not the unity of a single formation existing every where, but the unity of one system of formations, deposited on a scale of vast extent, and subsequently subjected to the same influences, whether conservative or destructive. In no other country, over an equal area, is the geological structure, so simple and uniform, and in no other does it so decidedly constitute the whole into one natural region."*

The external configuration of this valley, presents in its water courses great dividing lines, which no less than the mountain ranges of other more strongly marked countries, serve to establish its various divisions. Thus, at the northeastern extremity of the valley within the United States, we find the Alleghany river, taking its rise amid the Appalachian mountains in the state of New York, and pursuing a southwesterly direction, somewhat in the line of the great chain of lakes. The greater part, however, of the waters of the valley, after pursuing a southeasterly and southwesterly direction, as in the cases of the Missouri and Ohio rivers, find their way into the Gulf of Mexico, by means of the Mississippi river, which may be looked upon as the great outlet for the waters of the whole southern section of the valley which bears its name.

The medical topography of this region is as various as the difference in geological formation, subsequent deposits, and character of surface can render it, and consequently each separate locality has its peculiar topography, and its consequent diseases. Thus along the borders of the Mississippi river, are seen extensive alluvial deposits of great depth, reclaimed from the water, and terminated by bluffs. The most extensive of these is the American bottom, opposite St. Louis, in Illinois. This bottom has an average width of about five miles, and is nearly one hundred miles in length. This bottom consists generally of prairie, abounding in sloughs, ponds, lakes and bayous, which are filled in the spring, but become partially dry in midsummer and autumn, presenting a rank and luxuriant vegetation, to the influences most likely to generate miasma, and it is consequently peculiarly liable to autumnal intermittent and remittent fevers. A continued residence in the midst of malaria, has a tendency to render the inhabitants, to a certain degree, proof against its action in the form of fever, but they bear the evidences of its effects in their sallow and illy developed persons. Many of the inhabitants of this bottom are dwarfish and shrivelled, and are easily detected by their languid air, and sallow hue. The redemption from a watery dominion of this great alluvial region, "offers," says Dr. Drake, "to the engineer and the physician, problems in which the public have a deep and varied interest."

There can be no doubt that geological formation exercises an important

* Drake's Treatise, p. 28.

influence over disease. In proof of this, the reader of Dr. Drake's valuable treatise has but to compare the region of transition and secondary rocks, with the tertiary, in order to discover this influence, and to learn how intimately the geological character of a country is associated with its medical topography.

"In the region we have explored," says Dr. Drake, "it was found that as we advanced from south to north, there was a diminution in the prevalence of intermittent fever, which at the same time became more simple; there was also, but in a less degree, a diminished prevalence of remittent fever, and a gradually increasing tendency to assume a continued type. Although change of climate is a manifest cause of this modification, it is not, I presume, the only one, for we must also admit a telluric influence."

Another important agent in the modification of disease, which claimed the attention of Dr. Drake, was *climate.* In the interior valley, to which his investigations were confined, an extent of territory exists, which extends from the frigid zone upon the north, to the torrid zone upon the south, and which, consequently, contains every variety and modification of climate. But independent of mere latitude, there are a great variety of causes, which tend to produce decided alterations of climate in the valley of the Mississippi, among which may be mentioned its elevation above the level of the sea, the amount of its watery surface, and the presence of the Rocky mountains upon the west, and the Alleganys upon the east, bestowing upon it a complete intra-mountain temperature upon a vast scale. A large amount of information has been collected, but as yet, serious difficulties exist, in the absence of a sufficient series of long continued and extended observations, towards even an approximation to so difficult a subject, as the determining of the temperature of an extended and diversified country,

"In the west, from the cooling influences of the Cordilleras of Mexico, and the Rocky mountains, extending into the polar circle, and to the east, from a similar, though smaller, influence of the same kind, exerted by the Appalachian chain, from the latitude of 33° to 48° or 50° north, we know that the causes of equal mean temperature, cannot lie parallel to the lines of latitude, except for a certain distance in the middle of the valley, east of the Mississippi, as they approach the Appalachian mountains, they must bend to the south, west of that river, as they ascend the great inclined plane, they must curve in the same direction, and on reaching the Rocky mountains, must, of necessity, extend along their slopes, rising gradually, as the latitudes lessen, but not attaining the summits of these mountains, until we come within the tropics."

It is generally supposed that the settlement of a country, by clearing away its forests, and exposing a large portion of the surface of the earth to the influence of the sun's rays, has a decided influence in modifying its mean temperature, but such has not been the result in the valley of the Mississippi. On the contrary, it appears from thermometrical observations extended through one-third of a century, during which there has been a large increase in its population, that no change has taken place in the mean temperature of this valley.

A much more important element in climate, than either mean temperature or extremes in temperature, considered in connection with disease, is *humidity*.

"In our climatic geography, there are five great regions, which may be presumed to differ widely from each other in their absolute quantities of atmospheric vapor, and also in the dew point complement. They are, *First*, the Gulf of Mexico; *Second*, the region west of the Mississippi river; *Third*, the regions east of that river, between the gulf and lakes; *Fourth*, the lakes themselves; *Fifth*, the Arctic regions. Let us consider them in succession.

"1st.—*The Region of the Gulf.*—The atmosphere resting over the gulf, and its coasts, and the estuaries and deltas of its rivers, is constantly near the point of saturation; although from its high temperature, a large amount of vapor is necessary to that condition. Thus, the mean annual temperature of the thirtieth parallel, is seventy degrees, at which point, the quantity of vapor, which, according to the table, is necessary to the saturation of a cubic foot of air, is 0.8693; while at the thirty-ninth parallel, where the mean annual heat is 53°, the amount of vapor required to saturate the same quantity of air is only 0.5074. Hence, around the gulf, there is not only an impregnation of the atmosphere, nearly up to the point of saturation, but the absolute quantity of vapor is great. The dew point is always high, and its complement small. In every season of the year, the loss of a few degrees of temperature, is sufficient to cause the condensation of vapor, and render the air moist. When the wind blows over the interior of the continent from the gulf, it brings with it this great amount of vapor; and coming into colder climates, which have a lower dew point, the atmosphere is at first made damp, then hazy, and at last rainy. During the winter, the heat of the gulf keeps up, while that of the inland regions becomes greatly reduced; and in the spring, the winds from the former have their vapor condensed in passing over the latter, and hence the copious rains of that season. At midsummer, currents from the gulf are still passing to the north; but the air over the continent is so hot, that it can receive and retain much of their vapor, in addition to what it already possesses. In autumn, the continental atmosphere is cooled, and then the southern currents send down, in the form of rain, a liberal quantity of their vapor. Hence, there are vernal and autumnal floods in our rivers. If the Gulf of Mexico were filled up, the winds from that region would have a high temperature, with a low dew point, and would shed upon the interior but little rain—the condensation from the difference of temperature, would not reach the dew point.

"2d.—*Region West of the Gulf and the Mississippi.*—The great inclined plain west of these waters, stands in opposition to the region of the gulf. Its southern latitudes are as warm as those of the northern curve of the gulf; and even for ten or fifteen degrees further north, the summers are almost as hot as those of the latter region. The temperature is such as would admit of a high dew point; but the surface does not afford water for copious evaporation; and the air seldom approaches the state of saturation—is generally capable of receiving more vapor, and feels dry. The mountains to the west, are equally deficient in sources of vapor, and their low temperature causes the precipitation in the form of snow and frost, of so great a quantity, that the atmosphere over them has a dew point still lower than the atmosphere of the plain. When the winds of the gulf traverse that region, much of their vapor is required to bring its atmosphere to the point of saturation, and less is left to be precipitated in the form of rain or dew. On the eastern margin of the plain, near the Mississippi river, this is not the case, for the evaporation from the broad and watery trough of that river keeps up the atmospheric vapor; but on advancing toward the mountains, the quantity of vapor becomes so small, that it remains uncondensed during the minimum temperature of the summer night, and dew does not form. In autumn, however, when the temperature sinks still lower, saturation is reached, and vapor is then deposited in the form of hoar frost.

"These facts disclose to us the cause of the dryness, and the drying quality of our west and north-west winds. The absolute quantity of their vapor is small, much less than, at their temperature, they are capable of containing; and hence, when they roll over the eastern half of the valley, they take from it a large quantity of water. By the vulgar, their coldness is supposed to be the cause of their drying power, and hence they speak of freezing things dry; but if they came with the same small, absolute quantity of vapor, and had a high temperature, their drying power would be far greater. The winds which possess this power in the highest degree, are those which blow from a southern, sandy desert. They come with a low dew point, while their heat gives them a capacity for sustaining one much higher.

"3d.—*Region East of the Mississippi, between the Gulf and Lakes.*—The geological, hydrographical and botanical conditions of the region conspire in affording more vapor to the atmosphere, than the region beyond the Mississippi. The south-west winds which traverse it, come from the gulf, much oftener than those which traverse the more western plains. Finally, the north-east winds come almost saturated with vapor, afforded by David's Strait, the Gulf of St. Lawrence, and the northern Lakes. Under such circumstances, its atmosphere is of necessity more humid, has a higher dew point, and a complement of shorter range, than the region to the west of the Mississippi, between which and the region of the gulf, it may be regarded as holding an intermediate position.

"4th.—*Region of the Lakes.*—Here is abundance of water, but the temperature, compared with that of the gulf, is low; and the absolute quantity of atmospheric vapor is, of course, much less than along the shores of the gulf. Hygrometric observations have not yet determined the relative number of degrees in the dew point, of these two different regions, lying thirteen or fourteen degrees of latitude apart. At Toronto, the elastic force or tension of vapor, from observations every two hours for two years, was found to be 0.259 in., and the mean annual temperature for the same years was 44° 8. Now, by a reference to the table of tensions, we find that vapor of this temperature, when the air is saturated, has a tension of 0.313 in., showing that the atmosphere around the lakes, taking the year throughout, does not approach the dew point.

"At Hudson, nearly thirty miles south of lake Erie, Professor Loomis found the complement of the dew point for two years to be 8° 10′, while at Toronto it is about 5° 25′—difference 2° 85′, in favor of Hudson, which is what might be expected from their relations to the lakes.

"From the table of Professor Loomis, it appears that the month of April has the least vapor, compared with what, from its temperature, the atmosphere might contain. The complement of the dew point of that month, is 12° 80′; that of December, which has the least, is 4° 95′. Of the seasons, spring is the dryest, winter the most humid; summer and autumn are intermediate, and differ but little from each other. Two observations were made daily by Professor Loomis, one at nine, A. M., the other at three, P. M. The difference between them was, for the year, 5° 2′. The greatest difference was in spring—the least in winter.

"5th.—*Arctic Region.*—A reference to the general table of mean temperatures, and the table of this section, will show that the actual amount of vapor which can at any time exist in the atmosphere, within the polar circle, is very small. In the latitude of thirty degrees, where the mean temperature of the air is seventy degrees, it requires 0.8693 grains troy, to saturate a cubic foot of air. Under the fortieth parallel, which has a mean heat of fifty-one degrees, 0.4757 grains are required for saturation; at the seventieth degree of latitude, where the average annual is but five degrees, the required amount is only 0.0872 grains. In popular language, the vapor of the atmosphere is nearly frozen out. Still, in those regions, one of the chief inconveniences, experienced on shipboard in winter, was the humidity of the apartments. The atmosphere has no capacity for receiving the exhalations from the lungs and skin, which, being condensed against the walls, by re-evaporation, maintained the air at the dew point, while every thing without, had its moisture congealed and deposited. When the wind blows from that region, it does not, however, reach the more southern latitudes, in such destitution of vapor, for in traversing Hudson's bay, and a countless number of lakes, its temperature is raised, and it imbibes a great additional quantity of vapor."

The influence of moisture in the generation of miasm is well known, and in the late epidemic of Asiatic cholera, in the Mississippi valley, it was uniformly observed to linger about those places noted for their quantity of moisture, as the low damp situations by the sides of streams, or where the atmosphere was more highly charged than usual with aqueous vapor. Professor Davis, of Chicago, in an able paper on this subject, has given many conclusive proofs of the dependence of the spread of this fearful epidemic upon moisture, either contained in the atmosphere, incident to the position where the disease made its attack.

Apart from those circumstances produced by geological and climatological phenomena, there are physiological considerations, connected with the inhabitants themselves, which must not be lost sight of in an examination of this region. One of the most important of these physiological considerations, is the great diversity in the origin of the inhabitants, and the consequent diversity of their offspring by intermarriage, from which it follows, "that the world has never before witnessed such a commingling of races. Those of England and the Atlantic states, the most complete of modern times, bear no comparison with ours, and if we ascend to the earliest historic period, no case of equal complexity is met with. The Roman empire, it is true, was greatly compounded, it was, however, an assemblage of distinct nations, between which there was but little, and in many cases, no social nor even commercial intercourse. It was an aggregate, ours is a living compound, as yet in the forming stage. Three out of five varieties of the human species, with all the important races which belong to one—the Caucasian or overruling element—cannot fail in the end to give a new physiological and psychological development."*

But a small proportion of this voluminous work is devoted to the examination of particular diseases, and this is necessarily confined to the class of intermittent and remittent febrile affections. Dr. Drake has the materials for a second volume, which will be devoted to the examination of disease, which we hope will soon be placed before the medical public. In the meantime, the influence of the present work, cannot but be in the highest degree beneficial to the cause of medical science, and through it to that of humanity. If no other end is attained than that of drawing the attention of medical practitioners to these causes, which are silently operating in their midst, in modifying diseased action, and which have been hitherto too often overlooked, its great benefits can hardly be overestimated. No physician, and especially no country practitioner in the valley of the Mississippi, should be without this work. Its facts are carefully selected, its deductions accurately drawn, and an entire freedom from prejudice or preconceived theories characterizes the whole volume.

* Drake, p. 646.

